# Editorial: Epstein-Barr Virus-Associated T/NK-Cell Lymphoproliferative Diseases

**DOI:** 10.3389/fped.2019.00285

**Published:** 2019-07-10

**Authors:** Shigeyoshi Fujiwara, Hiroshi Kimura

**Affiliations:** ^1^Department of Allergy and Clinical Immunology, National Research Institute for Child Health and Development, Tokyo, Japan; ^2^Division of Hematology and Rheumatology, Department of Medicine, Nihon University School of Medicine, Tokyo, Japan; ^3^Department of Virology, Nagoya University Graduate School of Medicine, Nagoya, Japan

**Keywords:** EBV-associated T/NK-cell lymphoproliferative diseases, chronic active EBV infection (CAEBV), hydroa vacciniforme-like lymphoproliferative disorder, severe mosquito bite allergy, extranodal NK/T-cell lymphomanasal type (ENKTL), aggressive NK-cell leukemia (ANKL), EBV-associated hemophagocytic lymphohistiocytosis (EBV-HLH), systemic EBV-positive T-cell lymphoma of childhood

In the current 2016 version of the WHO classification of tumors of hematopoietic and lymphoid tissues, four disease entities are included as EBV-associated T/NK-cell lymphoproliferative diseases (EBV-T/NK-LPDs), namely systemic EBV-positive T-cell lymphoma of childhood, chronic active EBV infection (CAEBV) of T- and NK-cell type (including the systemic form and the two cutaneous forms, hydroa vacciniforme-like LPD and severe mosquito bite allergy), aggressive NK-cell leukemia (ANKL), and extranodal NK/T-cell lymphoma, nasal type (ENKTL). In addition, EBV-positive nodal peripheral T-cell lymphoma is included as a provisional entity. Although EBV-associated hemophagocytic lymphohistiocytosis (EBV-HLH) is not included, this syndrome is also characterized by non-neoplastic proliferation of EBV-infected T or NK cells.

EBV-T/NK-LPDs share some epidemiological, clinical, and pathophysiological properties, including geographical distribution almost restricted to East Asia and Central/South America. Multiple entities of EBV-T/NK-LPDs can be diagnosed in a single patient and one entity of EBV-T/NK-LPDs may evolve into another during the clinical course, suggesting a common mechanism in the pathogenesis, including genetic predisposition. However, the pathogenesis of EBV-T/NK-LPDs is largely unknown and therapy for most of these diseases is difficult and tends to depend on hematopoietic stem cell transplantation (HSCT). This Research Topic is intended to summarize recent progresses in the field of EBV-T/NK-LPDs and to provide a platform for discussion on their enigmatic pathogenesis and the development of novel therapeutic strategies.

Kimura and Fujiwara provide an overview of EBV-T/NK-LPDs, describing the definition, characteristics, and current therapies of these diseases. They also list unsolved questions concerning EBV-T/NK-LPDs and highlight the area where more investigations are required.

As Kim et al. note, the diagnosis of EBV-T/NK-LPDs is difficult because of their unusual clinical presentation and discrepancies between clinical and pathological findings (e.g., aggressive clinical course without apparent morphological atypia in neoplastic cells). In a comprehensive review of each entity of EBV-T/NK-LPDs, these authors describe characteristic clinical features, histology, immunophenotype, and molecular findings of the diseases that are essential for their correct diagnosis and proper management.

In a comprehensive review on CAEBV, Arai points out that the pathophysiology of CAEBV has two facets, inflammation and neoplasm. Although the molecular mechanism of EBV-induced T/NK-cell proliferation and survival has not been elucidated, she describes recent evidence suggesting pivotal roles for the NF-κB and JAK/STAT pathways. She suggests that therapies targeting these pathways may be effective against both the inflammatory and neoplastic features of CAEBV and may improve the result of HSCT via resolving disease activity before transplantation.

Ai and Xie review the literature on EBV-T/NK-LPDs reported from Chinese mainland, one of the few areas where these diseases are endemic. Although the general clinical picture of CAEBV appears similar between China and Japan, including poor prognosis without HSCT, they imply some differences, including higher incidence of lymphadenopathy and interstitial pneumonitis and lower incidence of hypersensitivity to mosquito bite in Chinese mainland as compared to Japan. However, they note that EBV-infected cell type has been determined only in a small percentage of patients in China and therefore the statistics of EBV-T/NK-LPDs must be interpreted carefully.

ANKL is a rare malignant lymphoproliferative disease of mature NK cells typically with the large granular lymphocyte morphology. It occurs mainly in younger adults and has very poor prognosis. Ishida contributes a minireview discussing recent molecular and clinical issues associated with ANKL. Next-generation sequencing of ANKL cells reveals frequent somatic mutation in genes involved in the JAK/STAT pathway and those associated with epigenetic gene regulation, suggesting the possibility of identifying a novel molecular target for therapy.

Harabuchi et al. contribute a comprehensive review on ENKTL. Especially, they summarize basic findings on ENKTL, starting from their own discovery of EBV DNA and proteins in tumor cells and including recent characterization of various cytokines, chemokines, and microRNAs that are expressed in association with EBV infection of T/NK cells and could be therapeutic targets.

De Mel et al. summarize recent progresses in genome-wide gene expression profiling (GEP) on EBV-T/NK-LPDs. GEP reveals upregulation of the JAK/STAT and NF-κB pathways that promote cell survival and proliferation in ENKTL. It also shows that upregulation of survivin and deregulation of p53 inhibit apoptosis in ENKTL and CAEBV. Furthermore, GEP identifies a number of possible therapeutic targets, including immune checkpoint molecules, CD38, and elements of the JAK/STAT pathway.

Tanita et al. describe a 21-year-old male patient with EBV-positive γδT-cell LPD who exhibits low T and NK-cell numbers, low T/NK-cell proliferative response, and deficiency in STAT3/5/6 phosphorylation following stimulation with cytokines. Whole exome sequencing identifies a hemizygous hypomorphic mutation of the *IL2RG* gene (c.C982T, p.R328^*^) that may be responsible for his immunodeficiency. This result suggests the possibility that CAEBV can develop on the basis of unidentified primary immunodeficiency.

Ishimura et al. describe a male case of CAEBV with a hypomorphic mutation of the *SH2D1A* gene (c.G7T, p.A3S). They also describe two male cases of CAEBV/EBV-HLH with a hypomorphic variant of the *XIAP* gene (c.1045_1047delGAG, p.E349del) and a female case of CAEBV with the same *XIAP* variant in a heterozygous state. These cases suggest that the two genes responsible for X-linked lymphoproliferative disease may be involved in the pathogenesis of EBV-T/NK-LPDs in a fraction of patients.

In a perspective article, Yachie highlights the value of flow-cytometric analysis of circulating lymphocytes in the diagnosis of EBV-T/NK-LPDs. He indicates that the expansion of HLA-DR^+^TCRγδ^+^ cells and that of HLA-DR^+^CD56^+^ cells are a diagnostic marker suggestive of hydroa vacciniforme-like LPD and severe mosquito bite allergy, respectively. Expansion of CD8^+^CD5^dim/negative^HLA-DR^high^ cells is a specific marker for the diagnosis of EBV-HLH. Flow-cytometry can also demonstrate monoclonality of EBV-infected T cells by revealing the expansion of a cell population expressing a particular TCR Vβ gene.

Sawada and Inoue describe a unified treatment strategy for EBV-T/NK-LPDs that is composed of the step 1 (immunochemotherapy), step 2 (multi-drug chemotherapy) and step 3 (allogeneic HSCT). They analyze a single-institution experience of planned (i.e., not emergent) transplantation (*n* = 63) for the treatment of CAEBV and show that reduced-intensity conditioning (RIC) is superior to myeloablative conditioning (MAC) with the 3-year overall survival of 90.7 ± 4.0% (*n* = 54) for the former and 66.7 ± 15.7% (*n* = 9) for the latter. Thy also use the unified strategy for the treatment of EBV-HLH; majority of cases require only the step 1 or the steps 1 and 2.

Sato contributes an opinion article that mainly summarizes recent preclinical studies on molecular target drugs for the treatment of EBV-T/NK-LPDs, including the anti-CC chemokine receptor 4 (CCR4) antibody mogamulizumab, the proteasome inhibitor bortezomib, HDAC inhibitors, mTOR inhibitors, and JAK inhibitors. He also mentions on the recent progress in organizing a nationwide registry of EBV-T/NK-LPD in Japan.

To conclude, the recent technical innovation in genomic research has begun to provide critical novel findings in both the host and virus genetics of EBV-T/NK-LPDs, providing new insights into the etiology and pathogenesis of the diseases. This trend will accelerate the development of improved therapeutic strategies for EBV-T/NK-LPDs including novel chemotherapy.

## Author Contributions

SF and HK participated in the editorial process of the Research Topic and co-wrote the editorial.

### Conflict of Interest Statement

The authors declare that the research was conducted in the absence of any commercial or financial relationships that could be construed as a potential conflict of interest.

